# Note from the editors: *Eurosurveillance* contributor survey results

**DOI:** 10.2807/1560-7917.ES.2020.25.45.2011121

**Published:** 2020-11-12

**Authors:** Eurosurveillance editorial team

**Affiliations:** 1 European Centre for Disease Prevention and Control (ECDC), Stockholm, Sweden

In January 2020, we ran an online survey to inform the future development of *Eurosurveillance*. We invited readers, authors and reviewers to share their views and expectations on journal content, features and engagement, e.g. outreach activities. Thank you to the 177 people who took the time to respond, as well as to the seven authors/reviewers who participated in in-depth interviews. We herewith share a brief summary of the results; percentages are given, and the full results and total numbers for percentages are available in the supplement.

The online questionnaire contained 27 closed and three open questions, and was available from 9 January for a period of 6 weeks. Structured interviews took place between 29 January and 21 February 2020, coinciding with the emergence of the coronavirus disease (COVID-19) pandemic. The survey was advertised through the journal website, social media (Twitter and LinkedIn) and editorial board members and colleagues. Both elements of the survey indicate that *Eurosurveillance*’s contributors and audience are overall satisfied with the quality of the journal and the work of the editorial team. *Eurosurveillance* is appreciated and seen as a credible and reliable source of important public health information and research, and is being used for policy-making and action. Similar findings were recently noted in an independent evaluation of the European Centre for Disease Prevention and Control (ECDC) [1], who funds and publishes the journal.

## Survey respondents

The majority of respondents were female (59%) and individuals aged 36 to 45 years represented the largest age group (28%). Only 18% were younger than 36 years of age and very few were below 26 years old. Respondents worked in many different countries, mainly within Europe, and 42% self-identified as public health practitioners or field epidemiologists. The majority read the journal regularly; nearly half (48%) read two to four articles published by *Eurosurveillance* each month. A considerable proportion of respondents not only read the journal, but were also authors (47%), reviewers (13%) or both (41%).

## Content attributes and use

With respect to content, half of the respondents rated the ‘overall quality’ of articles above average and 38% found them excellent. ‘Reliability’ of articles was rated even higher: 46% of respondents rated it excellent and total favourable (above average and excellent) ratings were 88%; ‘completeness’ of articles was rated slightly less favourably (79%). Only one respondent each found ‘reliability’ and ‘completeness’ below average. The attribute ‘understandability’ was rated as excellent by 44%, above average by 42% and average by 15%; none of the responders found it below average. Taken together this leaves only few respondents indicating room for improvement.

A further set of answers confirmed that respondents found the journal relevant or even very relevant for their work (85%) and that a large proportion of survey participants (36%) use articles for public health action, such as public health interventions (18%), other decision-making (10%) and policy-making (8%).

## Website features and engagement

Nearly half of the respondents (44%) rated their overall experience with the website above average, while 29% think of it as excellent and 21% as average. The individual questions on layout and features received unfavourable responses by very few respondents, with the search function having the most unfavourable responses.

Many of the respondents were not fully aware of or do not make use of the full array of *Eurosurveillance*’s outreach activities. In terms of social media, 16% were aware of our LinkedIn page, but 47% do not use it or are unaware of it; 45% of survey respondents were aware of our Twitter feed but do not use it, while 22% of survey participants use it. However, a large proportion of respondents (41%) mentioned that they read the journal’s collections.

## Open questions and in-depth interviews

The open survey questions and in-depth interviews with authors and reviewers demonstrated that contributors appreciate the support they have received from the *Eurosurveillance* team. Respondents and interviewees indicated that the journal provides good service that matches their needs and that they appreciate the work of the editors in contributing to improved article quality. However, they also expressed an interest in increasing the speed of processing times and reducing waiting times before publication. There were differing views with respect to processing times for articles, which some indicated had room for improvement and others described as excellent. Quick processing times for rapid communications received particular praise. The scope of the journal was also mentioned by interviewees as one area that could be more clearly defined.

## The way forward

We appreciate all of the input received via the contributor survey and interviews, which is helpful and relevant for day-to-day operations and strategic considerations. Our future focus will be on retaining the characteristics that contributed to respondents’ overall satisfaction and improving in areas that were flagged. Further, as the way that the survey was advertised may have led to a positive selection bias, we will also pay attention to the areas found to be average by a comparatively high proportion of survey respondents (such as ‘completeness’) or that were flagged as unclear in the interviews (such as ‘scope’), and strive to improve these aspects. Ongoing discussions with our editorial board and publisher may identify potential new future goals, in addition to our primary goal to remain a credible, authoritative and relevant source of sound public health information and evidence for decision- and policy-making, with focus on Europe.

## Supplementary Data

1503000 Supplement
