# Supplementary material for: Note from the editors: Eurosurveillance contributor survey results
Source: Euro Surveill. 2020 Nov 12;25(45):2011121. doi: 10.2807/1560-7917.ES.2020.25.45.2011121 (PMC7667633; doi:10.2807/1560-7917.ES.2020.25.45.2011121)
Supplement: Supplement [file 2011121-Supp.pdf]

REPORT

Eurosurveillance satisfaction survey

Sabrina Nothdurfter
Sabrina.Nothdurfter@ecdc.europa.eu

Table of Contents

Executive Summary ... 1

1. Background and aim ... 2

2. Methodology ... 2

    2.1. Quantitative method ... 2

    2.2 Qualitative method ... 3

3. Results ... 5

    3.1 Qualitative analysis: surveys ... 5

    3.2. Qualitative analysis: interviews ... 24

4. Conclusion ... 41

5. Annexes ... 43

Executive Summary

In January 2020, *Eurosurveillance* launched their second satisfaction survey in order to determine the level of its audience's satisfaction. The survey results will be used to determine the journal's future strategies. For data collection, qualitative and quantitative methods were used. This survey targeted the journal's readers, authors and reviewers, as *Eurosurveillance* was interested in determining the level of satisfaction from these different perspectives.

Quantitative data was collected via an online survey using the EUsurvey tool and was accessible online for a period of six weeks. The questionnaire consisted of 24 closed questions and 3 open questions. In total, 177 people responded to the survey. Additionally, seven telephone interviews with the journals' authors and reviewers were conducted over a period of one month to collect more in-depth information and to complement the data collected from the online survey. While the online survey focused on readers, authors and reviewers, the interviews only targeted the latter two.

Overall, the *Eurosurveillance* audience has a high level of satisfaction with the journal. Readers highly appreciate the journal's work and highlighted its importance for the field of public health. The journal's website and submission system were perceived as user-friendly and understandable. Moreover, respondents appreciated the editors' work and described them as supportive, helpful and available. Additionally, the results showed high contentment in terms of the quality of the different aspects of articles and the majority of respondents thought that article quality was improved after the editing process.

The main recommendation to the editorial team was to clarify the scope of the journal, as it raised many questions during the interviews. Moreover, the respondents suggested an increase in the capacities of the editorial team in order to avoid long article processing times, in particular for regular articles. It was also suggested that the journal consider a more structured peer-review process by providing specific questions to reviewers. In terms of the editing process, it was suggested that editors leave formatting to a later stage and provide authors with more time to respond to comments. Concerning article layouts, respondents suggested a change of colours, noting that nothing stands out from the articles; other suggestions were that large tables should be avoided and that a larger font should be used. Since this survey did not reach students, in particular the age group 18–25 years, increasing social media activity should be considered, as a younger audience is likely to be reached through social media channels.

Overall, the results of this survey were very positive and the *Eurosurveillance* audience is satisfied with the quality of the journal and the work of the editorial team. The identified areas of improvement provide a good basis for *Eurosurveillance* to define its future strategies.

1. Background and aim

In 2011, *Eurosurveillance* conducted a reader satisfaction survey that has contributed to informing its direction since then. One major outcome of this work was the launch of a new website in 2017. Given the time that has passed and the changes that have been made, *Eurosurveillance* conducted a satisfaction survey once again in order to determine the current views of readers, authors and reviewers and to inform their future direction. For data collection, qualitative and quantitative methods were used. This survey targeted the journal's readers, authors and reviewers, as *Eurosurveillance* was interested in determining satisfaction levels from these different perspectives. The online survey was launched on 9 January 2020 and was available for a period of six weeks. The seven in-depths interviews were conducted from 29 January to 21 February 2020.

The overall objectives of this survey were to determine levels of satisfaction with the journal's content, features and engagement, as well as to inform the journal's future strategy. To meet these objectives, the survey focused on three areas: journal content, journal features and journal engagement, such as outreach activities.

2. Methodology

2.1. Quantitative method

For quantitative data collection, an online questionnaire was developed using the EUsurvey tool. The questionnaire consisted of 27 questions divided into four sections: demographics, journal usage, journal website and specific questions for authors and reviewers. The questionnaire consisted of 24 closed questions and 3 open questions. The open questions were designed to give participants the possibility to freely comment in more detail on certain aspects. The online survey was advertised through the journal's social media channels (Twitter and LinkedIn) and website, as well as via emails to board members, who were asked to distribute the information to their networks.

The estimated time for responding was 10–15 minutes. Participants were asked to respond for themselves, not for their institutions. The survey respondents were also asked to read the privacy statement, aligning with the European Union (EU) regulations of processing personal data, and accept the terms prior to answering the questions. The survey was anonymous and participants were not asked for names or contact information. All the demographic questions were non-mandatory fields,

hence participants had the possibility to skip questions they were not comfortable answering. Moreover, the information from demographic questions was presented on an aggregated basis.

The collected data was imported from the EUsurvey tool into Excel. The data was sorted and cleaned before analysis. Afterwards the counts and percentages of each question were calculated. The answers for the open questions were imported into a Word document and were sorted and coded with a colour system for analysis.

2.2 Qualitative method

For qualitative data collection, seven in-depth interviews were conducted, targeting the journal's reviewers and authors, to collect more in-depth information and insights to complement the online survey findings. An interview guide was developed to structure the interviews in order to not miss any important information, as well as to ensure consistency, as the interviews were carried out by two investigators. The questions were based on participants' most recent submission or review to inform whether the processes went well or if there was need for improvement.

Different channels were used to invite the journal's authors and reviewers to participate in the interviews. Social media (Twitter and LinkedIn) was used for this purpose. Moreover, the website and the submissions system were used to advertise a call for interviewees. Additionally, board members were asked for recommendations of possible interview candidates who expressed their interest to participate. Once the targeted number of interviewees was reached, the interviewers were provided with all necessary information and times were arranged. In order to have a diverse representation of respondents, the people who expressed interest in being interviewed were selected according to their geographical location, professional background and sex.

Before the interview, all participants were asked to read the privacy statement that aligned with the EU regulations of processing personal data and the informed consent form. Additionally, they were asked to return the signed copy of the informed consent form prior to the interview. The duration of the interviews varied from 30 to 50 minutes. The interview guide was divided into four sections: demographics, submission/review, specific questions for authors and reviewers, and closing questions. All the interviews were tape recorded; participants were informed about this beforehand. The voice records were deleted after the transcripts were produced. The transcripts were sent out to participants in a Word document to give each interviewee the opportunity to read through it and comment on answers that were not transcribed accurately. After the participants' final approval, all the identifying information was removed from the transcripts and each of them was assigned a number in order to guarantee participants' confidentiality. All transcripts were imported to the

qualitative data analysis software OpenCode. Each document was analysed by assigning different codes to nearly each line and were then sorted and organised in a further step.

3. Results

3.1 Qualitative analysis: survey

In total, 177 people responded to the survey. The total number of answers varies throughout this section, as some of the questions had multiple answer options and others were specifically addressed to reviewers and authors.

Demographics

In order receive information about the characteristics of the *Eurosurveillance* audience, some demographic questions were asked.

What their sex is (n = 170; skipped: n = 7):

Answer options	Responses	
	n	%
Female	101	59
Male	69	41
Non-binary	0	0

The majority of respondents (59%) were female and 41% of respondents were male, while nobody identified as non-binary. Seven respondents did not wish to answer this question.

What age group they are in (n = 176; skipped: n = 1):

Answer options	Responses	
	n	%
18–25 years	4	2
26–35 years	29	16
36–45 years	50	28
46–55 years	44	25
56–65 years	43	24
≥ 66 years	6	3

The vast majority of the *Eurosurveillance* audience indicated to be 36 years and older. The biggest proportion of the audience (28%) is in the age group 36 to 45 years, followed by 25% in the age group 46 to 55 years. The next biggest groups are 55 to 65 year olds, represented by 24%, and 26 to 35 year olds, represented by 16%. The results indicate that *Eurosurveillance* does not attract very young readers, as only 2% of the *Eurosurveillance* readership are in the age group 18 to 25 years. One person did not wish to answer this question.

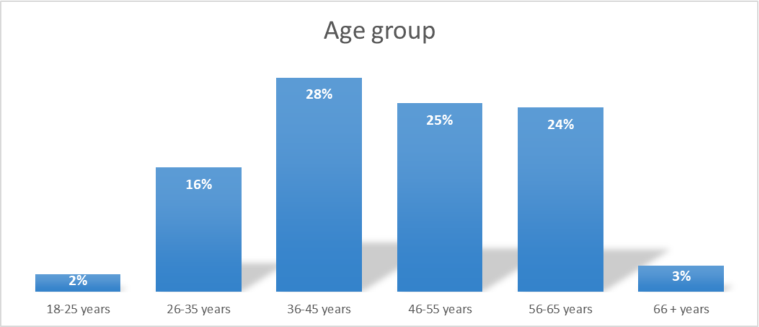

Which country they work in (n = 172; skipped: n = 5):

The answers for this question were grouped in three different categories to get a better overview of the countries representative in this survey. The survey respondents work in a broad range of countries, but mainly within Europe. The first group is *Eurosurveillance* countries, which are the countries that are represented in the Editorial board of the journal.

***Eurosurveillance* countries**	**Responses**	
	**n**	**%**
Austria	2	1
Belgium	8	5
Bulgaria	1	1
Canada	6	3
Croatia	10	6
Czech Republic	6	3
Denmark	5	3
Estonia	1	1
Finland	6	3
France	11	6
Germany	11	6
Greece	9	5
Hungary	2	1
Ireland	10	6
Italy	19	11
Luxembourg	1	1
Malta	2	1
Netherlands	6	3
Norway	2	1
Portugal	1	1
Romania	2	1
Slovenia	8	5
Spain	13	8
Sweden	16	9
Turkey	1	1
United Kingdom	3	2

The majority of respondents indicated that they worked within Europe and within the *Eurosurveillance* countries. Italy had the highest count with 19 respondents (11%), followed by Sweden with 16 respondents (9%) and Spain with 13 respondents (8%). Croatia, France, Germany and Ireland were represented by 6% each, followed by Canada (3%), the United Kingdom (2%) and Turkey (1%).

The other categories were European countries, which only included the two responses from Switzerland, and the last category, Non-European countries, including the United States (2%) as well as Australia, Morocco, Myanmar/Burma and Saudi Arabia, represented by 1% each.

**European countries**	**Responses**	
	**n**	**%**
Switzerland	2	1
**Non-European countries**		
Australia	2	1
Morocco	1	1
Myanmar/Burma	1	1
Saudi Arabia	1	1
United States	3	2

What type of organisation they work for (n = 196[a]; skipped: n = 2):

The majority of respondents work for national government authorities (37%), followed by research institutions (17%) and universities (11%). The minority of respondents work for non-governmental organisations (3%) and in the primary care sector (2%), while two respondents did not wish to answer this question.

**Answer options**	**Responses**	
	**n**	**%**
National government authority	72	37%
Research institution	34	17%
University	22	11%
Other	18	9%
Intergovernmental organisation	15	8%
Hospital	15	8%
Regional or municipal government authority	12	6%
Primary care	5	3%
Non-governmental organisation	3	2%

[a] This question had multiple answer options, therefore the total count differs from the previous question.

What occupation they have (n = 177; skipped: n = 0):

All of the survey respondents have replied to this question. The majority of respondents (42%, n = 74) are working as public health practitioners or field epidemiologists, followed by 8% each working as

public health policy-makers and in the area of infection control (n = 15), as well as 13 respondents (7%) working as infectious disease specialists. Nine of the respondents (5%) are working as microbiologists, followed by seven respondents (4%) working in laboratory research. Clinical medicine, diagnostics and general practice are represented by 2% each. Moreover, 2 % of respondents indicated they are currently trainees and students. The professional categories agriculture, behavioural science, media, nursing, paediatric medicine, teaching and tropical medicine are represented by 1% each.

**Professional categories**	**Responses**	
	**n**	**%**
**Public Health**		
Public health practice/field epidemiology	74	42
Public health policy	15	8
**Clinical Practice**		
Infection control	15	8
Infectious disease specialist	13	7
Clinical medicine	3	2
General practice	3	2
Tropical/travel medicine	2	1
Paediatric medicine	1	1
Other medical speciality	1	1
**Laboratory and Diagnostics**		
Microbiology	9	5
Laboratory research	7	4
Diagnostics	3	2
**Training/Education**		
Student	4	2
Intern/trainee	3	2
Teaching/education	2	1
**Related disciplines**		
Other	15	8
Behavioural science	2	1
Agriculture/food production	1	1
Nursing/other healthcare professional	1	1
Pharmacology/biotechnology	1	1
Veterinary medicine	1	1
Media	1	1

How many articles published by *Eurosurveillance* they have read in the past month (n = 177; skipped n = 0):

Answer options	Responses	
	n	%
One article	48	27
Two to four articles	82	46
Five to seven articles	15	8
More than seven articles	15	8
None	17	10

Nearly half of the respondents (48%; n = 82) have read two to four articles published by *Eurosurveillance* per month, followed by 27% (n = 48) who indicated they had read one article per month. Only 8% of the respondents (n = 15) specified they had read five to seven articles or more, while 10% of the respondents mentioned they had not read one article in the past month.

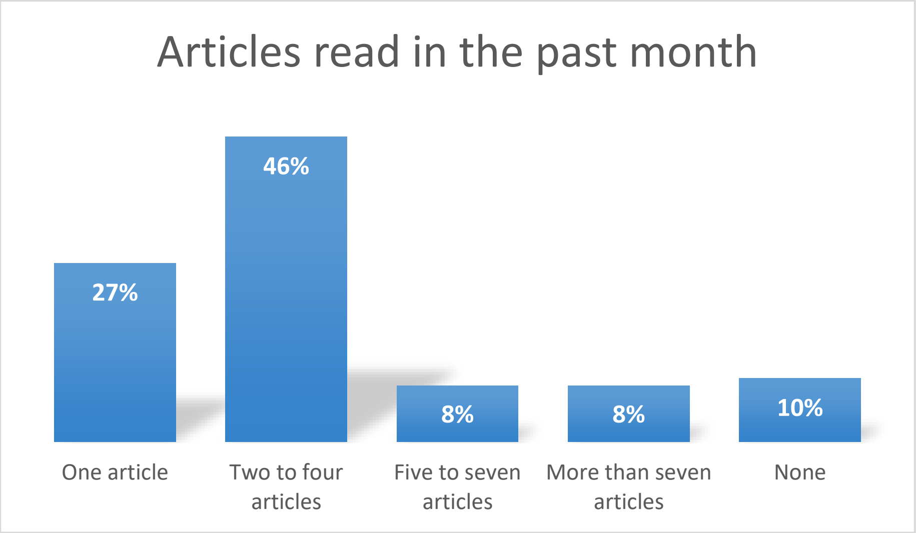

Journal articles

How they would rate the following aspects of *Eurosurveillance* articles (n = 177; skipped: n = 0):

Answer option	Excellent		Above average		Average		Poor		Very poor	
	n	%	n	%	n	%	n	%	n	%
Overall quality	68	38	88	50	21	12	0	0	0	0
Reliability	82	46	75	42	19	11	1	1	0	0
Completeness	67	38	73	41	36	20	0	0	1	1
Understandability	77	44	74	42	26	15	0	0	0	0

Survey participants were asked to rate the overall quality, reliability, completeness and understandability of *Eurosurveillance* articles. Half of the respondents (n = 88) rated the overall quality of articles above average and 68 of the participants (38%) as excellent. The other aspects were rated similarly and respondents were positive about them. The reliability of articles was perceived as excellent by 82 respondents (46%) and above average by 75 respondents (42%), while 19 respondents (11%) rated this aspect as average. When it comes to completeness of articles, the majority of respondents (41%; n = 73) mentioned this aspect to be above average, followed by 67 respondents (38%) ranking it excellent and 30 % (n = 36) as average. In terms of understandability of articles, 44% of the respondents rated this aspect as excellent and 42% as above average, while 26 respondents (15%) mentioned this aspect to be average. None of the respondents rated the overall quality and understandability as poor or very poor, although one respondent each perceived the reliability of articles as poor and the completeness as very poor.

How relevant the content of *Eurosurveillance* is for their work: (n = 177; skipped: n = 0):

Answer options	Responses	
	n	%
Very relevant	92	52
Relevant	58	33
Moderately relevant	20	11
Slightly relevant	5	3
Not relevant	2	1

More than half of the respondents (52%) have stated that the content of *Eurosurveillance* is very relevant to their work, followed by 33% who mentioned it to be relevant; 11% of the survey participants considered the relevance of *Eurosurveillance* for their work as moderate, while 3% of the respondents thought it was slightly relevant and 1% not relevant at all.

How they use the articles *Eurosurveillance* publishes (n = 496[a]; skipped n = 0)

Answer options	Responses	
	n	%
Research	115	23
General knowledge	113	23
Public health interventions	91	18
Teaching	50	10
Other decision making	49	10
Policy making	42	8
Clinical practice	22	4
Other	14	3

[a] This question had multiple answer options, therefore the total count differs from previous questions.

The majority of respondents (23%) indicated that they use the articles for research and general knowledge. The results indicate that a large proportion of survey participants (36%) use the journal for public health action, including public health interventions (18%; n = 91), other decision-making (10%; n = 49) and policy-making (8%; n = 42). Another 10% of the respondents mentioned that they use the journal for teaching, followed by clinical practice (4%), while 3% indicated that they use it for other purposes, but those were not indicated in a follow-up question.

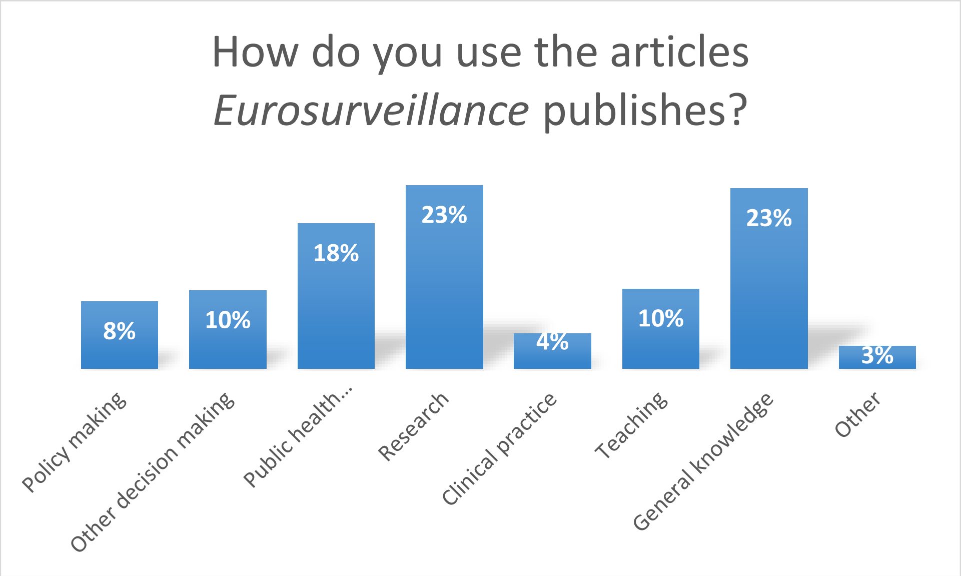

Are they reading articles that are not directly related to their field of work (n = 177; skipped: n = 0):

Answer options	Responses	
	n	%
Yes	112	63
No	65	37

The majority of the *Eurosurveillance* readership (63%) mentioned that they also read articles that are not directly related to their field of work, while 37% stated they only read articles related to their work.

Considering the latest *Eurosurveillance* article that they read, they rated the following elements (n = 177; skipped: n = 0):

Answer options	Excellent		Above average		Average		Poor		Very poor	
	n	%	n	%	n	%	n	%	n	%
Tables	63	36	83	47	30	17	1	1	0	0
Figures	65	37	83	47	27	15	2	1	0	0
Titles	65	37	76	43	35	20	1	1	0	0
Headings	65	37	72	41	39	22	1	1	0	0

In order to inform article quality, we wanted our readership to rate the tables, figures, titles and headings concerning the last *Eurosurveillance* article they read. Nearly half of the respondents (47%) rated the tables above average and 36% referred to them as excellent, while 17% think the tables are average. In terms of figures, 47% of participants rated their quality as above average and 37% excellent, while 15% of respondents referred to the figures as average. The titles were rated above average by 43% of respondents and excellent by 37% of respondents, whereas 20% ranked them average. In terms of headings, 41% of respondents thought their quality was above average and 37% ranked them excellent, while 22% indicated the headings as average. None of these four aspects was rated as very poor, although 1% each referred to all of them as poor.

For the elements rated as average, poor or very poor, how do they think these could be improved:
In order to receive feedback from the survey participants on why they rated the aspects from the previous question as average, poor or very poor, this open question was designed to identify areas of improvement. In total, 15 respondents commented on the reason for their ratings. Among those 15 comments, five referred to the tables' format and expressed suggestions on what could be done better. Two survey participants stated that less tables should be used, while another respondent suggested the use of a slightly larger text to make them more readable. Moreover, it was suggested by one of the respondents that the journal avoid large tables. One respondent suggested that table information could be visualised as graphs:

> *"Information in tables is sometimes not so useful for the reader; in some cases it could be visualised (graph)."*

Another respondent recommended changing the format of tables:

> *"Tables could be presented in a more appealing and more understandable way."*

Two of the survey respondents commented on the titles; one of them thought they could be more 'catchy', while the other respondent suggested that sample sizes not be included in the article title. The rest of the respondents did not comment on these specific aspects, but left recommendations for the general layout of *Eurosurveillance*. It was suggested that the journal use more than two colours, while one respondent did not like the layout at all, but did not comment on how this could be improved. The other survey participants were fine with the layout of the articles and had a very neutral opinion about it:

> *"Nothing specific. I marked average as they are consistent with the quality of other high-impact journals."*
>
> *"Fine as they are, but not better than other journals."*

Journal website

How often do they visit the *Eurosurveillance* website (n = 177; skipped: n = 0):

Answer options	Response	
	n	%
Once a week	44	25
About 2–3 times a week	56	32
About once a month	37	21
About once every 2–6 months	22	12
About once every 7–12 months	11	6
Less than once a year	7	4

Nearly a third of the respondents (32%) stated that they visit the website 2 to 3 times a week, followed by 25% who mentioned that they visit the website once a week. Further, 21% of the respondents indicated that they do so about once a month, while 12% only visit the website about every 2 to 6 months. Only 6% of the respondents pointed out that they only visit the website once every 7 to 12 months, whereas 4% of the respondents visit it less than once a year.

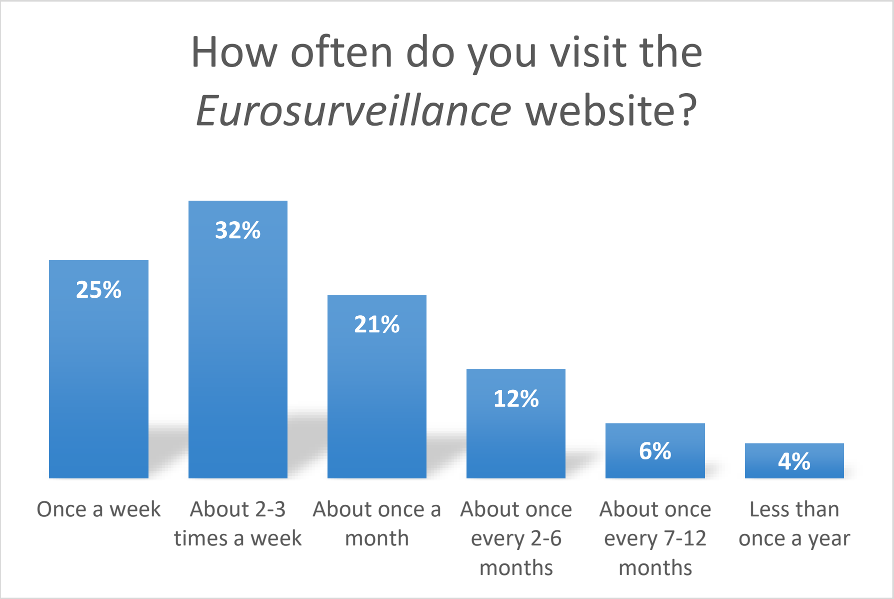

The respondents were asked to rate following aspects (n = 177; skipped: n = 0):

**Answer options**	**Excellent**		**Above average**		**Average**		**Poor**		**Very poor**		**Not applicable**	
	**n**	**%**	**n**	**%**	**n**	**%**	**n**	**%**	**n**	**%**	**n**	**%**
Overall experience with the website	52	29	77	44	37	21	2	1	1	1	8	5
The website's layout	51	29	73	41	41	23	2	1	2	2	8	5
The website's navigability	53	30	72	41	41	23	2	1	1	1	8	5
The website's user-friendliness	49	28	73	41	45	25	2	1	1	1	7	4
The website's search tool	44	25	61	34	44	25	5	3	1	1	22	12
The usefulness of search alerts	35	20	49	28	38	21	2	1	1	1	52	29
The usefulness of citation alerts	37	21	47	27	36	20	2	1	1	1	54	31
The usefulness of PowerPoint figure and table downloads	51	29	62	35	30	17	0	0	2	1	32	18
The usefulness of email alerts and RSS feeds	41	23	56	32	28	16	0	0	1	1	51	29

Nearly half of the respondents (44%) rated their overall experience with the website as above average and 29% excellent, while 21% think of it as average. The responses referring to the layout, navigability and user-friendliness follow a similar ranking. The websites' layout, navigability and user-friendliness were rate above average by 41% of the survey participants, followed by 29%, 30% and 28% respectively rating these aspects as excellent. Only a small proportion of the respondents (1%) rated any of those aspects as poor or very poor, although 3% think of the website's search tool as poor. The usefulness of alert functions was not applicable for about one third respondents. In terms of citation alerts, 31% of respondents mentioned this aspect as not applicable for them. In terms of email alerts and RSS feed, 29% of the survey participants rated those aspects as not applicable, while 27% of the respondents think the usefulness of citation alerts is above average.

Reasons why the aspects of the previous question were not applicable

We were interested to know the reasons why aspects were not applicable for those who indicated this in the previous question. Only a few respondents mentioned that the website's layout, navigability and user-friendliness, as well as their overall experience with the website, were not applicable for them. Five respondents commented on why they marked the overall experience with the website as

not applicable and stated that that they do not access the website regularly or search for/become informed about new articles through other means.

> *"I base my* Eurosurveillance *article selection and reading on monthly alert emails, not via the website."*

> *"Colleagues send me article suggestions by e-mail."*

The website's search tool was pointed out as not applicable by 22 respondents (12%). In total, 14 survey participants commented on their reasons. Among those, eight of the respondents mentioned that they simply do not use this function and hence it is not applicable for them:

> *"I have not really used any of those so impossible to assess."*

> *"I have never used them."*

When it comes to the usefulness of search alerts, 52 survey respondents (29%) selected not applicable. In total, 37 respondents explained the reason why they rated this as such. Similar to the previous aspects, the majority of respondents (n = 26) said the main reason they chose not applicable was simply that they do not use it, while two respondents commented that they were not aware of the alerts:

> *"I didn't know email alerts were possible."*

> *"Not sure I know what it is or if I have used it."*

Whereas one participant stated they would rather search for articles on the website:

> *"Searching actively the Eurosurveillance website or searching for articles when needed."*

Citation alerts were indicated as not applicable by 54 survey respondents (31%). Out of those, 36 respondents commented on the reason why they did so. Here too the answers follow the same pattern, as 25 out of the 36 respondents mentioned that they simply do not use them or they did not know about them:

> *"I have never looked at/used it."*

> *"I'm not using citation alerts."*

The PowerPoint figure and table downloads are not applicable for 32 survey participants (18%) and the email alerts and RSS feed for 51 respondents (29%). The answers did not differ much from the previous aspects. Twenty-three respondents mentioned that they do not use and do not have the need for them, such as this respondent:

> *"'I access regularly the site, so I do not feel the need to have any email or RSS alert feature."*

Journal engagement

We have different means of reaching our audience. Respondents were asked to indicate their use and awareness of these (n = 177; skipped: n = 0):

Answer options	Use		Aware, but don't use		Not aware	
	n	%	n	%	n	%
LinkedIn page	29	16	83	47	65	37
Twitter feed	39	22	79	45	59	33
Twitter quizzes	7	4	67	38	103	58
Collections on our website	72	41	50	28	55	31
Scientific seminars at ESCAIDE	51	29	75	42	51	29
ECDC summer school workshops	25	14	97	55	55	31
Other conferences workshop trainings	37	21	72	41	68	38

Eurosurveillance has different means of reaching its audience and wanted to understand which of these are used by the audience. In terms of social media, 16% of the audience was aware of the journal's LinkedIn page, but 47% indicated that they do not use it even though they were aware of it and 37% mentioned they were not aware of it at all. In terms of Twitter, 45% of the survey respondents were aware of the feed, but do not use it, while 22% of the survey participants use it. Compared to that, only 4% of the respondents use the Twitter quizzes, whereas a large proportion (58%) was not aware of these. A large proportion of respondents (41%) mentioned that they read the journal's collections, followed by 28% who were aware, but do not use these. In terms of education, 29% of the respondents pointed out that they've participated in the scientific seminars at ESCAIDE, while 42% are aware of these, but had not attended. One plausible reason for this is that not all the readers attend the conferences where the seminars and summer schools are usually held; however, 29% of the survey participants mentioned that they are not aware of these. In terms of summer schools, 14% of the respondents indicated that they were not aware of them and 21% mentioned that they've attended other conference workshops/trainings. Here too, a large proportion of survey respondents were not aware of the ECDC summer school (31%) and other conferences/trainings (38%).

Reasons for using the indicated means of outreach

We specifically wanted to know why respondents use the indicated means of outreach. Thirty-six respondents commented on their reasons. Among these, eight respondents mentioned that social media channels, such as LinkedIn and Twitter, are a great way to retrieve information. Those channels were considered as a simple, easy and fast channel to distribute and communicate information:

"They are easy and require little additional effort on my part, in the era of information overflow, this works well."

"Useful to get reliable information."

"I use LinkedIn and Twitter to be up-to-date for subjects relevant to my job."

The scientific seminars at ESCAIDE and summer school workshops were appreciated by the respondents, as they were described to be informative and great networking opportunities:

"I had the opportunity to participate in the seminars and found them interesting and relevant."

"Excellent and an opportunity to network."

As previously discussed, a large proportion of survey respondents indicated that they use the collections and described them as well-organised and useful:

"Although very few, collections are very interesting and useful."

"The collections are quite useful to get into topics."

Any additional comments

The last open question was phrased broadly to give survey participants the opportunity to address topics that had not been picked up in the questionnaire, to make suggestions for improvement or simply to offer feedback. One of the survey participants suggested that the journal consider implementing a voluntary presubmission peer-review service for less-represented countries:

"Eurosurveillance *could consider a voluntary pre-submission peer review/mentorship programme for authors from less well-represented countries within Europe. Some* Eurosurveillance *reviewers may be willing to support this on a voluntary basis and it would increase the quality of the articles submitted from some of these countries."*

Two other respondents suggested broadening the scope of the journal and taking the following topics into consideration:

"Infectious disease, travel medicine and surveillance should not be limited to vaccine-preventable diseases, bacteria or common arbovirus. Neglected tropical diseases and

mycoses do exist, policy problems do exist (like drug access issues) and control strategies do exist, they deserve space in the journal."

"Publish more real surveillance studies again, too many microbiological studies, focus too wide outside Europe."

Some of the respondents did not have any suggestions for improvement but wanted to acknowledge the work *Eurosurveillance* is doing and underlined the importance of the journal for public health:

"I look forward to Eurosurveillance *to stay current with global health and developing issues of international health such as the current coronavirus outbreak."*

"It cannot be emphasized enough how very important it is that Eurosurveillance *does not charge a publishing fee while it keeps an open access policy at the same time."*

*"*Eurosurveillance *is the best journal in my field. It is the most useful and timely publication. It stands out as being unique in having the quality to compete with the top academic journals whilst being an applied and essential tool for practise of public health."*

Specific questions

The majority of the questions in the online questionnaire were addressed to the *Eurosurveillance* readership; as we also wanted feedback from authors and reviewers, specific questions were designed and asked at the end of the survey. Complementary information was collected during the in-depth interviews and will be discussed later in this report. We wanted to know who of the survey participants had collaborated with the journal as a reviewer or author. 54% of survey respondents indicated they had authored articles published in *Eurosurveillance*, 14% mentioned having reviewed manuscripts and 46% of respondents indicated that they had done both. This illustrates that 96 out of 177 survey participants had collaborated with the journal at least once.

**Answer options**	**Reponses**	
	**n**	**%**
Author	45	47
Reviewer	12	13
Author and reviewer	39	41

Author-specific questions

How they would rate the submission system's user-friendliness (n = 84; skipped: n = 0):

**Answer options**	**Responses**	
	**n**	**%**
Excellent	29	35
Above average	36	43
Average	16	19
Poor	2	2
Very poor	1	1

In 2013, *Eurosurveillance* implemented an online submission system; therefore, we wanted to know how our authors have perceived this system. Thirty-six out of 84 respondents (43%) ranked the submission system's user-friendliness as above average and 35 respondents (43%) as excellent, while 19% rated it as average. Only two respondents referred to the system as poor and one respondent claimed it to be very poor.

How they would rate the guidance provided by *Eurosurveillance* in terms of submissions (n = 84; skipped: n = 0):

Over half of the respondents (51%) rated the guidance provided by *Eurosurveillance* in terms of submissions as above average and 35% rated this aspect as excellent, while 13% thought of it as

average. Only 1% of the respondents mentioned that the guidance provided by *Eurosurveillance* was very poor and none of the respondents indicated it as poor.

Answer options	Responses	
	n	%
Excellent	29	35
Above average	43	51
Average	11	13
Poor	0	0
Very poor	1	1

How they would rate the support they have received from *Eurosurveillance* during the editing process (n = 84; skipped: n = 0):

Answer options	Responses	
	n	%
Excellent	42	50
Above average	25	30
Average	15	18
Poor	1	1
Very poor	1	1

Half of the respondents (50%) expressed that the support they received from *Eurosurveillance* during the editing process was excellent, 30% referred to it as above average and 18% thought of it as average. Only 1% of the respondents rated it as either poor or very poor.

Whether they think article quality is improved after the editing process (n = 84; skipped: n = 0):

Answer options	Responses	
	n	%
Yes	80	95
No	4	5

We wanted to know whether authors think the article quality is improved after the editing process and almost all of the respondents (95%) indicated that article quality was improved, while only 5% stated the opposite.

Reviewer-specific questions

Whether they reviewed articles for *Eurosurveillance* in the past 3 years (n = 51; skipped: n = 0):

Answer options	Responses	
	n	%
Yes	42	82
No	9	18

We wanted to know whether the respondents who indicated to have reviewed for the journal have reviewed manuscripts for *Eurosurveillance* in the past three years. Forty-two of the respondents (82%) expressed that they have reviewed within this timeframe, whereas only nine respondents (18%) mentioned their last review for *Eurosurveillance* was not within the timeframe of three years.

If they indicated yes, how many articles had they reviewed in the last 3 years (n = 42; skipped: n = 0):

Answer options	Responses	
	n	%
1–2	18	43
3–4	17	40
5 or more	7	17

43% of the survey respondents mentioned that they have reviewed 1 to 2 articles in the past 3 years, 40% said they have reviewed 3 to 4 articles and 17% stated that they have reviewed 5 or more articles for *Eurosurveillance* in the past 3 years.

How would they rate the quality of the articles sent for review (n = 42; skipped: n = 0):

Answer options	Responses	
	n	%
Excellent	1	2
Above average	25	60
Average	16	38
Poor	0	0
Very poor	0	0

This question was used to indicate how our reviewers perceive the quality of articles sent for review. More than half of the respondents (60%) rated the quality as above average and 38% referred to it as average. Only 2% of the survey participants thought the quality was excellent and none of the respondents perceived the quality as poor or very poor.

3.2. Qualitative analysis: interviews

Seven telephone interviews with the journal's authors and reviewers were conducted to collect more in-depth information to complement the online survey. The questions focused on their last submission/review to *Eurosurveillance*.

Demographics

Like in the online questionnaire, interview participants were asked demographic information to identify the interviewees' characteristics. In total, seven in-depth interviews were conducted to complement the collected data from the online survey. The age of respondents ranged from 30 to 52 years old. All of the respondents were working in the field of public health. The majority of participants mentioned that they work for public health agencies on a national level, and only one respondent indicated that they work at a regional level. One respondent stated that they work as a research assistant, three as (medical) epidemiologists, one as a microbiologist and two as Heads of Unit. Among the seven interviewees, four expressed that they have authored articles for *Eurosurveillance* and three mentioned that they have authored articles and reviewed for the journal. The number of manuscripts interviewees had submitted to *Eurosurveillance* varied and ranged from one to 11 submissions in the past 5 years, while the number of reviews ranged from one to eight in the past three years.

We wanted to know whether our collaborators receive our weekly table of contents alert to be informed about the latest publications. Four survey participants mentioned that they have set up this alert, while three stated that they do not use it. Among those, two respondents pointed out that they check the website for publications and one of them stated that they only look for specific topics and therefore search for articles manually:

> *"In the past yes I used to, not anymore, because I don't need an email to alert. I check the Eurosurveillance website myself on Thursdays, it's part of my routines."*
>
> *"I am searching for topic-specific-papers."*

Furthermore, it is possible to install topic-specific alerts—such as search alerts and citation alerts—on the *Eurosurveillance* website, and we wanted to know whether they are used by our collaborators or not. Only one of the seven interviewees mentioned that they have search alerts installed, as well as one respondent who reported they have the citation alert installed. The majority of respondents claimed not to be aware of these alerts, but acknowledged that these functions would be interesting and useful. In addition, two of the respondents stated that they will look into it:

> *"No, I was not aware of such alerts but that would be interesting, I will check."*

"My colleague told me that she tried to set up the alert via the Eurosurveillance *website, but did not find where to set up these alert messages."*

"I did not set up a citation alert, but it would be a good idea to do so."

Submissions/Reviews

Thinking about your last manuscript, what is your opinion about the submission system?

All of the seven interviewees overall had a very positive opinion on the submission system. All of them noted that the system is easy, clear and precise. None of the respondents had any difficulties or could remember any problems while using the submission system and they remembered that they did not spend a lot of time on submitting their manuscripts:

"I have used other submission systems and I think it was easier than most systems. So yes, I liked it."

"Really easy and I like the system, it has been a really good system for a long time. I mean, even in the past I never had any problems."

Additionally, the respondents referred to the submission system as user-friendly and noted that all the information they needed was easy to find:

"Basically, I have a good opinion, because technically the website was quite user-friendly and the whole process went really fluent. The work process was very quick in my opinion."

"I am used to other publication systems, but yours was easy to understand. There was nothing special and there wasn't anything I did not find."

Can you think of anything that could be done differently in the submission system?

Although all respondents were overall positive of the submission system, some of them had suggestions for improvement. One of the interviewees did not refer to the submission system itself but had recommendations for the formatting:

"I remember Eurosurveillance *sent back my manuscript, because the references have to be in square brackets instead of parenthesis. It is very easy to format that after you have done all the reviewing. If there is one thing for improvement it is to be lenient with the first submission and then rather ask people to format differently*

when you come further down after all the actual theory and everything has been sorted by the peer-review."

One of the respondents stated that filling in the affiliations was time consuming, as many of them were not included in the submissions system's dropdown:

> *"I noticed that you could have some suggestions made by the system already but some affiliations in French were not available from the system. It would be great if one could spend less time on this part of the submission."*

Moreover, another interviewee mentioned having had problems with submitting the revised version of a manuscript and found it rather complicated, but acknowledged that the editors were supportive when this issue was addressed:

> *"Another aspect that I found rather difficult was the submission of the revision: it was unclear where I should upload the revised manuscript and whether I should upload the original submission as well. It was rather complicated but I got a lot of support from the editors, they were very helpful."*

How would you describe the information we provide about what we are looking for in terms of submissions?

Four of the seven interviewees mentioned that the information provided by *Eurosurveillance* regarding what they are looking for in terms of submissions is clear to them:

> *"I think it is easy to find and clear. I know the journal very well and usually the descriptions provided are useful."*

Moreover, the respondents expressed that they are checking the website, as well as other published articles, to see whether their submission is within the scope or not. Two of the respondents stated that they are probably biased since they are within the target audience of *Eurosurveillance* and therefore know what the journal is looking for and are aware of what would be of interest and what would not:

> *"I think it is very clear, but it is also true that I am working in a Public Health Institution within the EU, so I might be a bit biased. I am not sure if I were working outside Europe if it would have the same perception, but from my point of view it is very clear."*

Although the majority of respondents considered the information provided to be clear, a few discrepancies were mentioned while addressing this question. One survey participant added that they

are sometimes surprised, as some articles published by the journal are not within the scope of *Eurosurveillance*. The other three respondents found discrepancies in the information provided and do not quite understand the scope of the journal, admitting that there are quite some uncertainties. Another interviewee mentioned that the relevance for Europe is not clear to them and needs to be further described, while another respondent noted that the editorial policy of *Eurosurveillance* is not clear and needs to be adjusted and written more clearly:

> *"I think the publication policy must be described more detailed. In order to avoid submitting the same subject if it is not in the scope of the journal."*

Is it clear in general what types of articles we are looking for and who our target audience is?

In terms of articles types *Eurosurveillance* is looking for, all interviewees noted that it is clear to them and they know what article formats to use for which topics. In addition, the author guidelines appeared to be clear and do not raise any questions. Some respondents already mentioned discrepancies in the previous question and elaborated in more detail why they perceived the scope as unclear. One respondent mentioned that both target audience and editorial policy are not clear and asked for more detailed information. Another respondent noted that a more detailed description on research articles would be helpful. Furthermore, one of the interviewees mentioned a conflict of interest:

> *"I think generally it is quite clear, but I think that in terms of rapid communication I want to address something. It is clear to me what they want, but that is something that we cannot always provide. If you have a good outbreak then we tend to think that we do not want to use a rapid communication because it might hinder us publishing the full article later on."*

Do you have any other suggestions about additional information that should be added or ways that current information should be communicated differently on our website?

Two of the respondents were absolutely satisfied with the *Eurosurveillance* website and do not think it is necessary to add information or communicate the information differently:

> *"No. I think the Eurosurveillance website is quite good."*

The other five interviewees provided suggestions on what information the journal should consider adding. They addressed issues with the editorial policy, uncertainties in choosing the right format, as well as discrepancies regarding the scope of the journal, especially the relevance criteria. Respondents mentioned it would be useful to add the following information to the website:

> *"Maybe the criteria for what is relevant."*

"I do not know, but I think when you work in infectious disease surveillance almost every single development that happens to different pathogens across the world is in some way relevant. Because I found it hard to understand how to fulfil the relevance criteria."

"Where you describe the research articles you are looking for, the description says you are looking for research articles that provide original results from studies on any aspects of communicable diseases, epidemiology, prevention and control. In my opinion, it would be great to see a more detailed description on what this exactly means."

Moreover, one respondent who identified as an author did not know where to send presubmission inquiries and mentioned that they prefer to know beforehand whether the article is suitable for the journal or not, but it was addressed during the interview that the editorial office can be contacted with an inquiry via email:

"I do not know if there is a form or something like that so the editors do not get too many emails. I do not know if there is a parallel system to send these types of questions. "

What is your opinion on the technical instructions we provide about the submission/review process?

None of the respondents gave negative feedback on the technical instructions provided by *Eurosurveillance* about the submission and review process. All of the interviewees were satisfied with them and noted that the instructions are clear, easy to find and that no difficulties were had:

"I think Eurosurveillance *perhaps is the easiest I have published with."*

"The instructions were very clear, it's important to read them before starting the submission as I had to make some changes at a later stage."

Another respondent thought it is great that *Eurosurveillance* is an open access journal, as it is possible to look at other papers before submitting a manuscript, which also eases the formatting process. The interviewees did not have any problems while uploading the different files as the information was detailed and easy to understand. Therefore, none of the respondents mentioned that anything is missing in terms of the technical instructions:

"No, not really. You always provided me with support during the publication process. If I haven't met the total standard you would even have helped me out. I think you are doing a great job. "

"It is quite clear. Even if there was something, it was probably minor and was clarified during the process."

Even though all of the respondents were satisfied with the presentation and information of the technical instructions, one participant suggested categorising the importance of the technical information in order to have a clearer view on what details to spend more time on:

"Maybe it would be useful to have a hierarchisation of the technical information. That would be very important. For instance some journals say that if you do not use this format the paper will be rejected by the system. This would be a good point to know, and to pay attention to these points."

How would you rate the novelty of articles on a scale from 0 to 5, where 0 is not novel at all and 5 is extremely novel?

Six out of seven respondents distinguished between rapid communications and regular articles. All of the seven interviewees rated the novelty of rapid communications with the highest score, while six of the respondents ranked regular articles compared to rapid communications on a lower scale, mainly with the score three. The participants reasoned their choice that they all perceived rapid communications to be processed more quickly than other article formats, as they have to be published as they come, whereas regular articles have much longer processing times which creates a backlog:

"For rapid communications 5 which makes Eurosurveillance quite valuable for public health. For regular articles I would say 3 because it may take quite a long time to get them published and by the time these articles are published you don't feel that the topic is so novel anymore so these articles become a bit "old" when it comes to the data presented."

"5 because of the timely publications of the outbreaks. This is both in terms of novelty and timeliness for outbreaks. For the other articles, the process is so long that the articles lose their novelty and timeliness."

How would you rate the timeliness of articles on a scale from 0 to 5, where 0 is not timely at all and 5 is extremely timely?

Similar to the previous question, three of the survey respondents differentiated between rapid communications and regular articles:

"For rapid communications 5 because the data are usually really new and helpful. For regular articles it would be 3. In my field (vector-borne diseases) I think Eurosurveillance has been really helpful in publishing timely information e.g. for Ebola, MERS."

The other four interviewees perceived *Eurosurveillance* publications as timely and did not distinguish between the different types of articles. Both acknowledged the importance of *Eurosurveillance* in the field of infectious disease:

> *"As the focus of the journal is surveillance and outbreaks, I think* Eurosurveillance *covers the most recent developments in the area."*

> *"All right, I think* Eurosurveillance *is very timely. But then you need to be in the infectious surveillance field and I think that is why* Eurosurveillance *succeeds in the field, because it manages to be fast."*

Do you have any suggestions on how this could be improved?

Four respondents wanted to address suggestions for improvements in terms of timeliness and novelty of *Eurosurveillance* articles. All of them wished for faster processing times and a reduced waiting time until publication. Although all of the interviewees mentioned that they are aware this is difficult to achieve, as they know the *Eurosurveillance* editorial team is small and does not have endless capacities. One suggestion was to limit the submissions by a clearer definition of the scope of the journal. Another interviewee proposed enlarging the editorial board and increasing the number of editors, thereby increasing capacities in order to minimise processing times. Another idea was to be more selective and reject more papers to decrease the backlog:

> *"I wish it would go faster, but I am not involved in the editorial process and I cannot see where things can be improved. In my view, it has changed over the years, but* Eurosurveillance *used to publish almost everything and that created a large backlog. I think they are rejecting more papers nowadays, but also receive more submissions. However, of course the proportion of accepted articles needs to be balanced with the number of editors you have."*

> *"I think it is possible, but I realise it is difficult, because you receive a lot of papers. It might take up to a year with some papers to get accepted and I do not know whether that is something that can be improved. Clearly, it would be nice, but I understand it is not the easiest thing to do. I would commend definitely the rapid communications, because you manage to be very timely and provide topics, which are of interest as they come."*

Reviewer-specific questions

Four of the respondents have not only authored articles for *Eurosurveillance*, but have also reviewed for the journal. In order to collect more specific information regarding the quality of articles, guidelines and reviewers comments were addressed.

How would you rate the quality of the latest article you received as a reviewer?

Respondents were asked about the latest article they reviewed for *Eurosurveillance*. Only one of them noted that the manuscript received for review was of good quality:

> *"I remember it was a good paper. It has been a while and I think that I got enough information regarding what you require from my review so it was ok. I mean, I did not have any problems. My perception is when I receive a reviewer request from you is, that I expect the quality to be good."*

The other three respondents stated that the last manuscript they reviewed was not of good quality, but they also admitted that this explicitly applies to the latest manuscript and not to all the articles they have reviewed for the journal:

> *"I was rather surprised and disappointed by the quality of the latest article I reviewed for* Eurosurveillance*; the subject was quite interesting but unfortunately the authors did not give enough substance; as a reviewer, the lower the quality of the article, the more time you need to spend to explain to the authors all your comments and provide guidance on how the article can be improved. This was just 1 of the 8 articles I reviewed and unfortunately it wasn't good."*

> *"I think that was between moderate and poor. It was fairly well written and I think it followed all the guidelines in terms of abstracts and structure. However, the content was not very fascinating."*

Does the information you are given upon acceptance of the review and the journal's website provide you with sufficient information regarding what you need to consider during review?

All of the respondents mentioned that they were satisfied with the information they received from *Eurosurveillance* upon the review of an article:

> *"It is helpful to know the general rules. Usually each paper is different so you can't be very specific but in the general the rules are there and they are helpful."*

> *"I think the instructions are fine."*

One of the interviewees could not recall whether he received the reviewer guidelines or not. Only one of the respondents mentioned that there were some uncertainties and they wished for a more structured reviewing process, as it was perceived that the co-reviewer did something completely different. It was suggested that the journal have a more standardised review in order to get better results from the review and the editors should address more clearly what needs to be looked at:

> *"There is a section for the editors and information or a structure could be added there. For example,* Eurosurveillance *wants to know whatever. I mean you have the checklist at the end, but more specifically, to what the editors want to know: Should I publish it and why or why not? Also, what are the obstacles for it? I mean I try to summarise it the best way I can in the narrative, but probably a structure would help if* Eurosurveillance *wants to have a standardised format in which they receive the thoughts of a reviewer."*

Did you read the latest article you have reviewed when it was published?

As most of the reviewers were not satisfied with the quality of the latest article they have reviewed for *Eurosurveillance*, they recommended to reject the manuscript. Therefore, this question was generalised to get an idea of whether they read the articles once they are published or not. All of the respondents noted that they read the articles or at least scan through them, as they are interested in whether their comments have been considered:

> *"Usually I try to read to see the corrections made."*

Additionally, all four respondents mentioned that the article quality improved after reviews and authors' revisions:

> *"I often notice great improvement in terms of language and clarity."*

> *"I think the overall quality of the article has improved."*

One of the interviewees stated that the quality did improve, but not significantly, as the paper was already of good quality when it was sent out for review.

Do you think your comments were considered and adequately taken into account in the published version of the article?

Three of four respondents thought their comments were adequately taken into account upon publication of the manuscript. One of the interviewees mentioned that they distinguish between major and minor comments and that they only expect the major comments to be addressed in the

manuscript. Another respondent mentioned that it is absolutely okay to not accept all the comments made, but that it is important to at least refer to them in the limitations of the article:

> *"For the minor comments, I don't expect them all to be taken into consideration."*

One respondent mentioned that it varies from article to article and added that there are journals that follow the provided comments after review more closely:

> *"There are journals where my comments are more closely followed. My feeling is that* Eurosurveillance *gives authors more free way not to answer or not to respond to reviewer questions. When an editor has a question this of course needs to be addressed. For reviewer's comments, you have more freedom to ignore them. It is quite frustrating, when you spend a day or two on the review, and then you see that almost nothing has changed."*

Author-specific questions

Thinking of your latest submission, what did you think of the reviewers' comments?

Six out of the seven respondents referred positively to the reviewer comments they have received, as they perceived them as helpful, relevant, constructive and clear:

> *"There are journals where my comments are more closely followed. I think they were really helpful. Sometimes when you write something you are in the topic, so it is hard to see thing that can be improved. It was good to have that external view and it helped us to improve the overall paper. I appreciated it."*

> *"They were helpful, they made sense and we managed to adjust and the article was in the end more concise and to the point."*

One interviewee mentioned that they only received one comment from one reviewer and thought both of the reviews differed, but was overall positive about the comments received:

> *"We had two reviewers and both of them provided supportive opinions. However, the first reviewer had only one comment and the second one was maybe an epidemiologist, because he/she focused on the epidemiological part of the manuscript, but all of the suggestions and remarks, which were minor comments helped to improve the credibility of some points in the manuscript. Overall, I think they contributed to the improvement of the manuscript."*

One respondent was not very positive about the reviewers' comments, and perceived them as not well thought out. Moreover, the interviewee added that it seemed the reviewers did not spend a lot of time of reading the manuscript:

> *"For the last manuscript, they were very easy to answer, because my impression is the reviewers did not read it quite carefully. That is good for me, because then it is easy to answer them and you do not have to invest so much work in them. So, from a selfish standpoint that is good. I am not convinced the comments were of greatest quality."*

The majority of the respondents perceived the comments as clear and constructive, and also noted that the editors were helpful in explaining the reviewers' comments and giving good instructions on what to do:

> *"I liked that the editor told us what they thought after review what we have to make and what we should make and how we should make them. Yeah the editors were helpful in the peer-review process."*

> *"It was clearly somebody who had taken the time, gone through it, and thought about it."*

One author referred to them as not being very constructive and noted the impression that the reviewers did not understand the methodology used for that particular article:

> *"They did not understand the methodology. Therefore, the questions showed they did not understand of what we did. However, this is helpful, because then we know that we cannot assume the others cannot understand what we meant. There was nothing in the comments where we were thinking that is a good point, we have not thought about this."*

What do you think of the article layout?

Six out of seven respondents had positive feedback on the article layout and mentioned that it is nice, clear and sober. One interviewee who generally liked the layout had one minor suggestion to make it even better:

> *"They could be a bit better when they spend more on a graphical editor to redo all the figures. I think* Eurosurveillance *would go from 9.9 to 10 in layout when they have someone to redo all the figures."*

Another respondent referred to the article layout as unique and admitted that they prefer the online version of the journal to the print version:

> *"I like the online layout, because you can go to different sections if you want to. I am probably old fashioned, but I like the article in the pdf or printed out. I would prefer if the abstract would stand out more. I think the only difference between the abstract and the text is the different colour of the font in the abstract. That is something I do not like so much."*

Furthermore, one respondent also mentioned that they prefer the print version and wished that the *Eurosurveillance* issues would be downloadable in one single pdf, as it would be more convenient to have the entire issue compiled in one file:

> *"You could also send the issues in one pdf with a single mail because now you can only get single articles as pdf but not the entire issue. It makes it easier to get more cohesion when you have one subject, like a theme special issue because then you can have an editorial that discusses the articles together in one pdf with one layout."*

What do you think of the figures and tables?

The respondents were overall positive about the figures and tables, but three respondents have mentioned that they could be slightly improved.

> *"In the pdf version of our paper, one of the figures is slightly smaller than on the website, but it is not disturbing at all, as you can download them separately."*

> *"I do like the figures and there is nothing I can add to it. When it comes to the tables, I find the font size is a little small in comparison to the cell. It is like tiny numbers in a large cell. This seems disproportionate."*

How did you experience the review and revision process, including interaction with the editors?

All of the respondents perceived the review and revision process as very positive and rated the communication with the editors as very good. The authors mentioned that they always got prompt replies when they approached the editorial team.

> *"Overall we had a really good communication."*

> *"Everything went fluently, because we always got prompt replies from the editors."*

Some of the respondents added that their last submission was a rapid communication and there the communication had to be fast. It was also addressed that the submission system is useful for tracking

the status of the paper. As already discussed in the section "Submission/Review", two of the interviewees mentioned that they wish the process would go faster, as it can take quite some time. One of the respondents suggested to at least inform authors about the estimated publishing time:

> *"Maybe informing the authors from time to time, giving them some updates on when they can expect to have their article published – these would be helpful."*

How did you experience the editing process, including interaction with the editors?

All seven respondents were satisfied with the editing process and stated to have liked working together with the editors. They were satisfied and considered the editing process as fast and smooth. The majority of the interviewees addressed that the editors were helpful and responsive. They received clear instructions from the editors on how and what to do. In case of uncertainties and requests from authors, they received prompt replies from the editors with explanations:

> *"I think it very clear and it puts you through the process very smoothly in a short time. I think it works."*

> *"I do think it is always good. I appreciate the editing process, because I think it is good work there. I think it is very constructive and it is very communicative way of working together."*

> *"Everything was fast and everyone was helpful. Whenever I sent an email, I got an answer the same day every time."*

Are there any aspects that need improvement and what are your suggestions for that?

Although all the authors were positive about the editing process, they also expressed negative experiences during the editing process and provided suggestions on what could be done differently. One of the respondents mentioned that it would be easier to sometimes just call the editors in order to discuss uncertainties, as it is not always simple to explain what you mean in a comment box. Another interviewee mentioned that they received a lot of comments and did perceive this as frustrating:

> *"The editors spend a lot of time to improve the quality of the paper and they make many comments which may seem too much compared to other journals but the quality increases and this is a positive aspect."*

Moreover, one respondent perceived the amount of comments as stressful as well, especially as they were addressed in the final stage before publishing and there was a

tight timeframe to answer them. Despite that, the respondent was positive about the editing process:

> *"What was really surprising for me, that we got so many remarks and questions immediately before publishing and had relatively short times to give answers to those questions. It would be a little bit more convenient to get the questions a little bit before together with the reviewer's comments. It would be easier to answer."*

Have you ever had a paper rejected by *Eurosurveillance*?

Five out of the seven respondents mentioned that they have had their paper rejected by *Eurosurveillance*. Among these, one interviewee mentioned that they had only co-authored a paper that was rejected. For three of them, it was clear why their paper was rejected and they did understand the reasons given. One respondent mentioned that the data was simply too old while another one noted that it used a mixed method approach that was outside the scope of *Eurosurveillance*. The third respondent also mentioned that their method did not fit the profile of the journal. Two of the respondents stated that the rejection reasons were not clear for them; one of the respondents said it was absolutely unclear and the email that informed about both rejections was standardised so it was impossible to draw conclusions:

> *"They were rejected, but with no clear understanding of the reason, because we got a standardised response. It is always the same sentence and it is not informative. In fact, we do not know if it is the subject, like it is irrelevant for* Eurosurveillance *or if it is not timely."*

This respondent also added that it would be helpful to know the reason for rejection, as it is hard to understand what the decisions are based on, e.g. do they only read the abstract or go through the paper? The respondent mentioned trying to find explanations for the rejections, but that it was only possible to make assumptions. Another respondent explained a similar situation:

> *"I think I have been rejected twice. The other paper did not even make it to the review process and we did not understand why. We have seen a similar article coming out a couple of months later on the same topic. We did not understand why this was superior to ours, but that might be just author's view of frustration."*

Did you have any further interaction after receiving the rejection with the *Eurosurveillance* editorial team?

All of the respondents who had their manuscripts rejected are still submitting articles to *Eurosurveillance*. One respondent stated that it is not a personal thing and if one manuscript does not fit into the scope of the journal it is fine; there are other journals to submit those to. The

respondent added that *Eurosurveillance* is a good journal and therefore they continue submitting the manuscripts that are within the scope. One interviewee expressed frustration about not knowing why a manuscript was rejected, although they continue to submit but with less enthusiasm:

> *"I continue to submit of course, but not with the same enthusiasm. For instance, as I do not have a response to my questions, I am wondering if it is really the topic of* Eurosurveillance*. Is it really important for them?"*

Closing questions

At present, *Eurosurveillance* focuses on quantitative research. We have received feedback that we should consider publishing outcomes from qualitative research, e.g. social sciences. What is your opinion about that?

Only one respondent thought it would not be a good idea to do so; the other six respondents reacted positively to this question. They thought it was a good idea to open up to qualitative research, although they raised some concerns. One concern was that it could divide the target audience, as *Eurosurveillance* strongly focuses on epidemiology and hence quantitative research:

> *"No, I don't think we should mix up things."*
>
> *"It would of course affect the audience."*
>
> *"I like that* Eurosurveillance *has this specific focus and broadening the focus may make* Eurosurveillance *lose this unique feature so I am not totally in favour."*

Another concern was that the editorial board would need to be enlarged, reviewers from the field of social science would be needed, as well editors that are specialised in this field. Broadening the scope of *Eurosurveillance* would therefore require adding additional resources:

> *"I am not sure if* Eurosurveillance *is ready to publish that, because. I mean you need some reviewers and specialists from social sciences. Moreover, it is not the same rule for publication and the format is very specific for* Eurosurveillance*."*
>
> *"More resources would be needed in the* Eurosurveillance *team – a new editor with expertise in social sciences. Another challenge would be finding reviewers from this field. If resources can be found, maybe yes, but the focus should really be on evidence-based interventions."*

One point the majority of the positive respondents were very direct about was that if *Eurosurveillance* opens up for qualitative research it has to be in the field of communicable diseases

and outbreaks, as this is the scope of the journal and its target audience wants to read about exactly these topics:

> *"I think qualitative research is even within my field in epidemiology important. It is important to expand and to sort of fill in the blanks like the experience of going through a TB screening can be very important to creating policy in addition to how much TB you find. I think we have to try to bring them both together within the field of infectious disease."*

> *"Social science is very specific, although we know that for Ebola or some epidemics like that it is really relevant. But I am not sure if* Eurosurveillance *is ready to publish that."*

> *"That would be supportive if this would be related to outbreaks. This is good.*
> *I would say if qualitative research adds to our understanding in public health, then I do not see the point why someone would not want to publish it. I do think there is a space for qualitative research, but I have not had the feeling so far, that* Eurosurveillance *is missing something."*

> *"It has to have a relevance to public health, infectious diseases and epidemiology. Or the social network aspects as well, like how the spreading of a disease has to do with a number of factors that we usually don't look at. That would definitely be interesting as long as it has a clear public health return, so that we can actually use it. That is one nice thing about* Eurosurveillance*, that it provides evidence for action."*

Some of the respondents did not have any concerns about whether the journal opens to qualitative research. They noted that is a good idea and that it is important to mix these methods, as they would fill in blanks and look at diseases from a different angle, providing additional knowledge that epidemiologists could also benefit from:

> *"I think that is important. I do not like that in the Public Health field that we think of quantitative and qualitative research are two different paradigms that don't really mix together."*

> *"From my perspective, I would be very interested in reading about these types of studies that approach our problems with different methodologies, because I would learn a little bit more about those."*

And now for the final question of the interview, do you have any other comments/suggestions that you would like to give to the *Eurosurveillance* editorial team?

With the last question, time was given to comment on issues that have not been discussed in the interview and for final feedback. Two respondents did have suggestions on what to add to the content of *Eurosurveillance*:

> *"I think it is a good idea that* Eurosurveillance *is open to other topics, so social science yes, but also among specific population, e.g. sub groups of populations when you have some specific vulnerable populations, such as migrants for example. I think it is a new issue for Europe. Maybe a population approach is also important and not only the disease approach (e.g. climate change, migrants)."*

> *"For me it would be important to see how* Eurosurveillance *would consider opening up to more laboratory related researches. Papers in which the laboratory result."*

The others gave positive feedback and show they highly appreciate *Eurosurveillance* and consider it as an important asset for public health in Europe:

> *"Is a very important part of European infectious disease prevention and control and I think it is important that* Eurosurveillance *keeps doing what it does."*

> *"I think the editorial team is doing a great job, I think this journal is very important for Europe and it covers a very important topic. The information covered by* Eurosurveillance *is not available in other publications so I don't think there is a particular need to change anything in right now. "*

> *"I really appreciate the paper and I do not have any additional things to add."*

> *"I think you do quite well. In general, I think* Eurosurveillance *does a good job. Overall, I am quite happy with it."*

4. Conclusion

Overall, the survey results show that the level of satisfaction with *Eurosurveillance* is high and respondents expressed that they appreciate the work the journal is doing, as it is an important asset for public health in Europe. The survey respondents mainly work within *Eurosurveillance* countries and within national government authorities and research institutions. Moreover, the majority of respondents work in public health practice and epidemiology. A large proportion of the respondents indicated that they use the journal for public health action.

The majority of the respondents perceived the journal's submission system as good, as it is user-friendly and easy to understand. In 2016, *Eurosurveillance* launched a new website and the results illustrate that the change was an improvement, as the majority of respondents rated different aspects of the website as above average. Moreover, the results have shown that the audience is satisfied with the editing process and interactions with the editors. Survey participants expressed a high level of satisfaction with the guidance provided by the editors and support from the editorial team during the editing process. The vast majority of respondents perceive that article quality improved after the editing process. The editors were described as helpful, responsive, available and supportive, and all the interviewees perceived the editing process as very good. The opinions on the quality of articles sent for review was also rated as good, although a third of the respondents think it is average. Additionally, the majority of interview respondents perceived the quality of the article they last reviewed for the journal as of poor quality, but added this only referred to the last one reviewed.

Despite the high level of satisfaction, the survey respondents also made interesting suggestions on improvements the journal could take into consideration. One of the main recommendation to the editorial team is to clarify the scope of the journal, as it raised many questions during the interviews. Moreover, the editorial team should look into the possibility of increasing their capacity, as the respondents expressed that they would like to have faster article processing times. However, it was acknowledged that this would be difficult to achieve due to the small number of editors working at *Eurosurveillance*. Shorter processing times would also lead to faster publishing, as this was another area of improvement identified, as for regular article the respondents did perceive the data as not as novel anymore. Rapid communications are excluded from these views, as they were rated as extremely time and novel with very short processing times.

It was also addressed that the editors should send specific questions to the reviewers, instead of only providing the reviewer checklist, as this would lead to better quality reviews. In terms of the editing process, *Eurosurveillance* should consider leaving formatting to a later stage and not upon the review of an article, as well as not have short deadlines to answer the comments. In terms of article layout,

the respondents suggested changing the colours, as nothing stands out from the articles, and it was addressed that large tables should be avoided and a larger font should be used.

In summary, it can be said that the *Eurosurveillance* audience is overall satisfied with the journal and the work of the editorial team is much appreciated in the field of public health. These results, including the identified areas of improvement, will help to identify the future directions of the journal and are a basis for changes that could be made to further improve the quality of the journal.

5. ANNEXES

Online questionnaire

(Demographics)

Question 1:
Are you?
□ Male □ Female □ Non-binary

Question 2:
How old are you?
□ 18–25 years
□ 26–35 years
□ 36–45 years
□ 46–55 years
□ 56–65 years
□ 66 years and above

Question 3:
Which country do you work in?

**List of European countries**		
Albania	Italy	Switzerland
Andorra	Latvia	Turkey
Austria	Liechtenstein	Ukraine
Belarus	Lithuania	United Kingdom
Belgium	Luxembourg	**List of other countries**
Bosnia and Herzegovina	North Macedonia	
Bulgaria	Malta	
Croatia	Moldova	
Cyprus	Monaco	
Czech Republic	Montenegro	
Denmark	Netherlands	
Estonia	Norway	
~~Faroe Islands~~	Poland	
Finland	Portugal	
France	Romania	
Germany	Russia	
~~Gibraltar~~	San Marino	
Greece	Serbia	
~~Greenland~~	Slovakia	
Hungary	Slovenia	
Iceland	Spain	
Ireland	Sweden	

Question 4:
What type of organisation do you work for? Please select all that apply.
1. Intergovernmental organisation
2. National government authority
3. Regional or municipal government authority
4. University
5. Primary care
6. Hospital
7. Research institution
8. Non-governmental organisation
9. Other

Question 5:
Please name the organisation you are working at.

Question 6:
Which of the following best describes your current occupation?
1. General practice
2. Clinical medicine
3. Paediatric medicine
4. Tropical/travel medicine
5. Infection control
6. Infectious disease specialist
7. Other medical speciality
8. Veterinary medicine
9. Nursing/other health care professional
10. Laboratory research
11. Diagnostics
12. Pharmacology/biotechnology
13. Microbiology
14. Behavioural science
15. Public health practice/field epidemiology
16. Public health policy
17. Teaching/education
18. Agricultural/food production
19. Media
20. Student
21. Intern/trainee
22. Other

(Journal usage)

Question 7:
How many articles published by *Eurosurveillance* have read in the past month?
□ None of them
□ One article
□ Two to four articles
□ Five to seven articles
□ More than seven articles

(Journal articles)

Question 8:
How would you rate the following aspects of *Eurosurveillance* articles?

	Excellent	Above average	Average	Poor	Very poor
Overall quality	□	□	□	□	□
Reliability	□	□	□	□	□
Completeness of information provided	□	□	□	□	□
Understandability	□	□	□	□	□

Question 9:
How relevant is the content of *Eurosurveillance* to your work?
□ Very relevant □ Relevant □ Moderately relevant □ Slightly relevant □ Not relevant

Question 10:
How do you use the articles we publish? Please select all that apply.
□ Policy making
□ Other decision making
□ Public health interventions
□ Research
□ Clinical practice
□ Teaching
□ General knowledge
□ Other

Question 11:
Do you read articles that are not directly related to your field of work?
□ Yes □ No

Question 12:
Consider the latest *Eurosurveillance* article that you read. Please rate the following elements of the article in terms of their understandability:

	Excellent	Above average	Average	Poor	Very poor
Tables	□	□	□	□	□
Figures	□	□	□	□	□
Titles	□	□	□	□	□
Headings	□	□	□	□	□

Question 13:
For those elements that you rated as average, poor or very poor, how do you think they could be improved?

(Journal website)

Question 14:
How often do you visit the *Eurosurveillance* website?
□ Once a week
□ About two to three times a month
□ About once a month
□ About once every two to six months
□ About once every seven to twelve months
□ Less than once a year

Question 15:
Please rate the following aspects:

	Excellent	Above average	Average	Poor	Very poor	Not applicable
Overall experience with the website	□	□	□	□	□	□
The website's layout	□	□	□	□	□	□
The website's navigability	□	□	□	□	□	□
The website's user-friendliness	□	□	□	□	□	□
The website's search tool	□	□	□	□	□	□
The usefulness of search alerts	□	□	□	□	□	□
The usefulness of citation alerts	□	□	□	□	□	□
The usefulness of PowerPoint figure and table downloads	□	□	□	□	□	□
Email alerts and RSS feeds	□	□	□	□	□	□

Question 16:
For those aspects of the previous question that you said were not applicable, please explain why.

(Journal engagement)

Question 17:
We have different means of reaching out to our audience. Please indicate your use and awareness of them.

	Use	Aware, but don't use	Not aware
LinkedIn page	◻	◻	◻
Twitter feed	◻	◻	◻
Twitter quizzes	◻	◻	◻
Collections on our website	◻	◻	◻
Scientific Seminars at ESCAIDE	◻	◻	◻
ECDC summer school workshops	◻	◻	◻
Other conference workshops and trainings	◻	◻	◻

Question 18:
For those means of reaching out that you are using, please tell us why.

Question 19:
If there is anything else that you would like to address, please comment on such here.

If you have **not** authored and/or reviewed articles for *Eurosurveillance*, please submit your survey now. If you have authored and/or reviewed articles for *Eurosurveillance*, please proceed.

(Author- and reviewer-specific questions)

Question 20:
Please indicate if you have authored and/or reviewed articles with us. Select all that apply to answer a few additional questions specific to these roles.
◻ Author ◻ Reviewer

(Authors)

Question 21:
How would you rate the submission system's user-friendliness?
◻ Excellent ◻ Above average ◻ Average ◻ Poor ◻ Very poor

Question 22:
How would you rate the guidance provided by *Eurosurveillance* in terms of submissions?
◻ Excellent ◻ Above average ◻ Average ◻ Poor ◻ Very poor

Question 23:
How would you rate the support you have received from *Eurosurveillance* during the editing process?
◻ Excellent ◻ Above average ◻ Average ◻ Poor ◻ Very poor

Question 24:
Do you think article quality is improved after the editing process?
□ Yes □ No

<u>Reviewers</u>
Question 25:
Have you reviewed articles for *Eurosurveillance* in the past 3 years?
□ Yes □ No

Question 25:
If yes, how many articles have you reviewed in the last 3 years?
□ 1–2
□ 3–4
□ 5 or more

Question 26
How would you rate the quality of the articles sent for review?

Interview guide

The following is an overview of the questions that will be covered during the in *Eurosurveillance* interview. We are sending them to you know so that you can give your responses some thought prior to the interview itself.

General questions

We will start with some demographic questions and then some general journal usage questions:

1. Can you please tell me your age?

- RESPONSE

2. Can you please tell me what country you work in?

- RESPONSE

3. What organisation do you work at?

- RESPONSE

4. What is your current occupation?

- RESPONSE

5. What type of collaboration do you have with the journal: are you a reviewer or author or both?

- RESPONSE

6. **[IF AUTHOR]** How many manuscripts have you submitted in the past 5 years?

- RESPONSE

[IF REVIEWER] How many manuscripts have you reviewed in the past 5 years?

- RESPONSE

7. Do you receive an alert that a new issue of *Eurosurveillance* has been published every Thursday?

- RESPONSE

8. Do you have other publication-associated alerts installed, for example topic-specific search alerts or citation alerts?

- RESPONSE

Submission system-specific questions

I would now like to talk about your experience with the submission system. Please think about your latest article that you submitted to or reviewed for *Eurosurveillance*.

9. Thinking about this last manuscript, what is your opinion about the submission system?

- RESPONSE

As you know, *Eurosurveillance* focuses on the epidemiology, surveillance, prevention and control of communicable diseases in Europe. It also publishes articles from outside Europe, but these articles have to have a public health relevance for Europe.

10. How would you describe the information we provide about what are looking for in terms of submissions?

- RESPONSE

11. Is it clear what types of articles we are looking for and who our target audience is?

- RESPONSE

12. Do you have any other suggestions about additional information that should be added to or ways that current information should be communicated differently our website?

- Response

13. What is your opinion on the technical instructions we provide about the submission/review process?

- RESPONSE

14. **[IF AUTHOR]** Is it clear what files need to be uploaded and in what format?

- RESPONSE

15. **[IF AUTHOR]** Is anything missing in terms of instructions

- RESPONSE

16. Do you have any suggestions about how we can improve on communicating technical information?

- RESPONSE

Now thinking more generally about the novelty and timeliness of the articles that *Eurosurveillance* publishes.

17. How would you rate the novelty of articles on a scale from 0 to 5, where 0 is not novel at all and 5 is extremely novel. Why?

- RESPONSE

18.How would you rate the timeliness of articles on a scale from 0 to 5, where 0 is not timely at all and 5 is extremely timely?

- RESPONSE

Reviewer-specific questions

Now I'm going to ask you some specific questions about the last article you reviewed.

19. How would you rate the quality of the latest article you received as a reviewer?

- RESPONSE

20. Does the information you are given upon acceptance of the review and the journal's website provide you with sufficient information regarding what you need to consider during review?

- RESPONSE

21. Did you read the latest article you have reviewed when it was published?

- RESPONSE

22. **[YES]** What did you think of the article quality after your review and the author's revision?

- RESPONSE

23. Do you think your comments were considered and adequately taken into account in the published version of the article?

- RESPONSE

Author-specific questions

Now I'm going to ask you some specific questions about the last article you submitted to the journal.

24. Thinking of your latest submission, what did you think of the reviewer's comments?

- RESPONSE

25. What do you think of the article layout?

- RESPONSE

26. What do you think of the figures and tables?

- RESPONSE

27. Do you have any suggestions on what could be done better in terms of article layout and figure/table formats?

- RESPONSE

28. Thinking again of your last article with us, how did you experience the review and revision process, including interaction with the editors?

- RESPONSE

29. Are there any aspects that need improvement and what are your suggestions for that?

- RESPONSE

30. How did you experience the editing process, including interaction with the editors?

- RESPONSE

31. Are there any aspects that need improvement and what are your suggestions for that?

- RESPONSE

32. Have you ever had a paper rejected by *Eurosurveillance*?

- RESPONSE

33. Did you have any further interaction after receiving the rejection with the *Eurosurveillance* editorial team?

- RESPONSE

Closing questions

34. At present, *Eurosurveillance* focusses on quantitative research. We have received feedback that we should consider publishing outcomes from qualitative research, e.g. social sciences. What is your opinion about that?

- RESPONSE

35. And now for the final question of the interview, do you have any other comments/suggestions that you would like to give to the *Eurosurveillance* editorial team?

- RESPONSE

Informed consent

Project title: *Eurosurveillance* satisfaction survey

Investigator: XXXX

Participant's name: XXXX

Eurosurveillance has launched a reader and contributor survey in January 2020. The survey questionnaire has been available on our website since 9 January 2020. In addition to that, we are conducting telephone interviews with selected journal contributors to ascertain in-depth information of their views about *Eurosurveillance*. We anticipate that the interviews and the survey results will provide us with input about our strengths and areas for improvement, as well as priorities for the future.

Please note the following:

- The interview will take approximately 30 to 40 minutes.
- You have the right to stop the interview at any time.
- The interview will be electronically recorded and a transcript will be produced afterwards.
- You will receive the transcript to enable you to correct any factual errors.
- The transcript of the interview will be analysed by the *Eurosurveillance* team at ECDC.
- Interview content or direct quotations from the interview that are made available in a final report or other reporting/communication outlets will be anonymised so that you cannot be identified, and care will be taken to ensure that information from the interview that could identify you will not be revealed.
- All or part of the content of your interview may be used in an anonymised form:
    - In a final report;
    - In/on other outlets, including but not limited to, the *Eurosurveillance* website and PowerPoints for oral presentations at conferences;
    - At feedback events, e.g. with our publisher and editorial board.

This consent form is necessary for us to ensure that you understand the purpose of your involvement and that you agree to the terms and conditions of you participation.

By signing this form, I agree that I have read the previous information, and I agree to participate to the interview and that my personal data is processed in accordance with the attached privacy statement.

Name of participant	Date	Signature

Name of investigator	Date	Signature
